# Analysis of the effectiveness of a non-governmental organization in supporting clubfoot clinic at a tertiary care center

**DOI:** 10.1051/sicotj/2015008

**Published:** 2015-06-05

**Authors:** Pulak Sharma, Rahul Verma, Ashish Gohiya, Sanjiv Gaur

**Affiliations:** 1 Department of Orthopaedics & Traumatology, Gandhi Medical College Bhopal India

**Keywords:** NGO, Clubfoot, Effectiveness

## Abstract

*Background*: Since December 2011, a non-governmental organization (NGO) has been associated with our clubfoot clinic. Debates related to the effectiveness of NGOs in clinical milieu have raged on for a long time. The aim of this study was to analyze the effectiveness of an NGO in supporting the running of a clubfoot clinic in a tertiary care center.

*Methods*: A descriptive, observational study was conducted from October to December 2014. The three main conceptualizations of effectiveness namely goals, resources and reputation were evaluated for this study, and to analyze them, we compared our treatment data with other published reports, and devised a ten-point questionnaire looking into the working of the NGO. This questionnaire was given to all parents (49) of children with clubfoot attending the clinic and also to an independent observer who was present at the time of patients’ interactions with the counselor. The significance of patients’ and observers’ response was tested by Wilcoxon matched-pairs signed-rank test.

*Results*: 138 cases with 228 feet were registered in the clubfoot clinic till the end of our study. The average number of visits by the patients was 6.67 and 69.47% of cases required tenotomy during the course of treatment. Of the 49 patients, 33 (67.35%) graded the role of the NGO as excellent, while the rest showed a good response; 28 observers (57.14%) responded as excellent. The average score of parents towards role of the NGO was significantly higher than the observer’s score.

*Conclusions*: The NGO associated with our clubfoot clinic successfully supported formal health care professionals.

## Introduction

Clubfoot has since long been an unsolved clinical challenge for the orthopedic surgeons. The problem is more serious in developing countries on account of late presentation, higher rate of dropouts from treatment, and superstitious beliefs attached to this congenital problem.

Our hospital is a tertiary care center which caters to a large number of patients with clubfoot. We run a clubfoot clinic at our hospital which operates twice a week, on Tuesdays and Fridays. Optimal functioning of the clinic to adequately meet patient demand requires appropriate harnessing of our assets, human and natural resources and capital. Government and private sector entities are not structured to meet all the demands by themselves. Many argue that the voluntary sector may be better placed to assess the needs of the poor and provide suitable services, and to encourage the change in attitudes and practices necessary for the best utilization of resources [1–3].

Non-governmental organizations (NGOs) include a wide variety of groups and institutions that are entirely or largely independent of government, and are characterized primarily by humanitarian or cooperative, rather than commercial objectives. However, the terminology may vary from place to place; in the United States they may be called “private voluntary organizations”, while most African NGOs prefer to be called “voluntary development organizations”. Although organizations such as universities or research institutes may be non-governmental, the term NGO refers principally to private organizations that pursue activities to relieve suffering, promote the interests of the poor, protect the environment, provide basic social services, or undertake community development [4].

Since December 2011, an NGO has been associated with our clubfoot clinic. The NGO provides full-time personnel for the purpose of counseling, record keeping, ensuring follow-up, and answering basic queries related to the treatment. It also provides study material to the patients, ensures compliance, and delivers braces free of cost to children at the completion of corrective cast application. As the local and institutional visibility of this NGO increased, concerns about its actual impact on the program arose. The NGOs’ self-proclaimed effectiveness is often doubted and considered ambiguous by many authorities. Furthermore, there is a lack of published evidence supporting the effectiveness of such NGOs in specialized clinics in India that can serve as a benchmark for others.

The aim of this study was to analyze the effectiveness of the NGO in supporting the running of the clubfoot clinic at our tertiary care center.

## Methods

A descriptive, observational study, using qualitative and quantitative data collection tools was conducted from October to December 2014, almost three years after the NGO first became associated with the clubfoot clinic at our tertiary care center. Three main conceptualizations of effectiveness – goals, resources, and reputation – were evaluated in this study [5].

The goal of the NGO to provide effective treatment to all clubfoot patients was evaluated by comparing the treatment data at our clinic with data from other published reports. To measure the other two parameters, a 10-point questionnaire looking into the working of the NGO was devised (Appendix I). This questionnaire was given to the parents of children with clubfoot attending the clinic who were at the stage of bracing, and to an independent observer who was present at patient interactions with the counselor. These observers were interns and final year post-graduate students who were posted in the clubfoot clinic. They were asked to observe two sessions per week. Both the parents and observers were required to complete the questionnaire independently at the end of the session, and provide their response to the questions on a scale of 1–10, with one being worst and 10 being the best response.

The 10-point questionnaire that was given to the parents and observers aimed to assess the resources utilized by the NGO in terms of the study material provided to the parents, the time given to each counseling session, the quality of braces provided, their role in ensuring adequate follow-up and skills in building empathetic relations, and their reputation among the parents. The results were graded as excellent if the total score of both the observer and parents was more than 85 out of 100, good if the score was between 70 and 85, and poor if the score was less than 70. The significance of responses from parents and observers was tested by using the Wilcoxon matched-pairs signed-rank test.

## Results

A systematic review of the records of the NGO revealed that a total of 138 cases with 228 feet were registered in the clubfoot clinic until the end of the study period. Male children were more commonly affected than female children, in a ratio of 1.88:1. Bilateral cases were most common followed by right-sided clubfoot (B/L:Rt:Lt = 86:27:25). Fifty-eight cases registered were less than six weeks of age. The total mean Pirani score at presentation was 5.6. The corresponding hind foot score and mid foot score were 2.9 and 2.8, respectively. Average number of visits by the patients was 6.67 and 69.47% of cases required tenotomy during the course of their treatment. Three weeks after tenotomy or the final cast, plaster was removed and a brace was applied. The brace that was provided by the NGO consisted of open-toe hightop straight-last shoes attached to a bar. For unilateral cases, the brace was set at 60–70° of external rotation on the clubfoot side and 30–40° of external rotation on the normal side. In bilateral cases, it was set at 70° of external rotation on each side. It was ensured that the bar was of sufficient length so that the heels of the shoes were at shoulder width. The parents were instructed that the brace should be worn full time (day and night) for the first 3 months after the last cast had been removed. After that, the child should wear the brace for 12 h at night and 2–4 h in the middle of the day, for a total of 14–16 h during each 24-hour period. We continued this protocol until the child was 3–4 years of age. Contact details of all the patients were present with mobile numbers wherever applicable. The diagnosis of each case, Pirani scoring at each visit, and other important details of treatment were also found documented.

The data of the patients registered with the clinic showed a decrease in the dropout rates from three out of 22 in first year to one out of 39 in second year, further improving to zero dropout in the third year. The average period of follow-up after the last cast was 12 months with a range from 6 to 18 months. Five patients needed postero-medial soft tissue release for insufficient initial correction; three of these children had presented after 1 year of age and two had syndromic clubfoot. Ten patients had a relapse during bracing; eight out of these 10 parents, used to remove the brace at night as they felt that the child was not able to sleep properly while wearing the brace. The remaining two parents discontinued the brace as they were not able to come to the clinic to change its size. It was only when they noticed the recurrence of the deformity that they reported back. These patients were managed by repeated corrective casts at weekly intervals. There was a success rate of 60% in these patients, with the remaining four patients requiring a la carte surgical intervention. At our clubfoot clinic, the overall success rate of correction of clubfoot by casting alone was 93.3%.

Forty-nine parents of the children attending the clinic and six independent observers were given the 10-point questionnaire. The average score of parents for the role of NGO was significantly higher than the observers’ score (*P* ≤ 8.889e^−6^ i.e. *p* < 0.001).

When assessing the responses of the parents and observers individually, 33 parents (67.35%) graded the role of the NGO as excellent, while the rest indicated a good response, whereas 28 observers (57.14%) responded as excellent. No parent or observer was of the view that the role of the NGO in the clubfoot clinic was bad (Table 1).

**Table 1.** Patient’s and observer’s response on role of NGO.

		Patient’s response		
		Excellent	Good	Total
Observer’s	Excellent	28	–	28
response	Good	5	16	21
	Total	33	16	49

Parents and observers gave the maximum score to Question 10 (reputation of the clinic). The minimum average score of 8.51 was given by parents to Questions 5 (record keeping) and 7 (help during hospital visit). The minimum score of 8.41 was given to Question 1 (availability of the counselor) by the independent observers (Table 2).

**Table 2.** Showing mean and standard deviation of patients and observer’s grading of 10 questions.

Question no.	Patient’s score	Observer’s score
	Mean ± *SD*	Mean ± *SD*
1	8.77 ± 0.71	8.41 ± 0.64
2	8.89 ± 0.65	8.57 ± 0.67
3	8.79 ± 0.73	8.47 ± 0.64
4	8.53 ± 0.74	8.51 ± 0.74
5	8.51 ± 0.82	8.51 ± 0.82
6	8.73 ± 0.57	8.63 ± 0.48
7	8.51 ± 0.79	8.63 ± 0.69
8	8.55 ± 0.74	8.55 ± 0.74
9	8.89 ± 0.55	8.79 ± 0.61
10	9.02 ± 0.62	8.79 ± 0.64
Total	87.22 ± 0.032	85.69 ± 3.83

While evaluating the significance of responses by parents and observers using the Wilcoxon matched-pairs signed-rank test, it was observed that the average response given by parents for the role of NGO was significantly higher than the response by the observers to the same question (*p* < 0.001).

## Discussion

Debates related to the effectiveness of the non-governmental organizations have existed for a long time. NGOs are often charged with strategic philanthropy and face increasing pressures to show measureable results. We evaluated the working of the NGO at our clubfoot clinic using a multidimensional approach, which eliminates all chances of being influenced by external factors.

Records were appropriately maintained for all the patients registered in the clinic and this was a definite asset for the clinic. The questions related to record keeping (Question 5) and the role in ensuring follow-up (Question 6) had a mean score of 8.52 and 8.73 from the parents, and 8.51 and 8.63 from the observers, respectively. Before the association of the NGO, it was common to miss the data recording of patients, owing to the large volume and limited working hands in the clinic. The NGOs’ association helped immeasurably in this aspect. Not only were the patients’ contact details maintained, but also Pirani scoring of all the patients at every visit was recorded. This helped in mapping the patients geographically over the region and in monitoring their treatment during the subsequent visits.

Losing patients to follow-up during their treatment was also a frequently encountered problem; this was alleviated through the counseling sessions provided by the NGO, focusing on the importance of regular follow-up. The NGO reminded each patient of the date and time of their next visit verbally in person or telephonically, and helped in controlling the inflow of patients by scheduling their visits in a way that the average number of patients visiting the clinic remained manageable for the attending doctors. This led to a greater satisfaction among the patients and further ensured their commitment to regular follow-up.

Counseling sessions before the beginning of treatment played a major role in building the confidence of the parents toward the treatment. The use of serial photographs of treated patients, distribution of study material about clubfoot, and the emphasis on the importance of regular and timely visits streamlined the functioning of the clinic. The response to Question 2 (duration of counseling sessions) clearly indicated that the time allotted for the counseling sessions was adequate both from the parents’ and observers’ perspective.

In terms of counseling skills, most patients received essential information and adequate support. The study material provided during the counseling sessions was in the local language and was pictorially supported. The questions related to the content of counseling (Question 3) and study material (Question 4) had a mean score of 8.79 and 8.53 from the parents and 8.57 and 8.47 from the observers, respectively. This clearly depicted a satisfaction on part of the parents as well as the observers. The reputation of the NGO among parents was given the maximum score as judged by both parents (mean score of 9.02) themselves and the independent observers (mean score of 8.79). Reputation in view of the effectiveness shows how favorably the organization is viewed by the parents and observers.

Of the children with clubfeet who presented to us, only 42% did so within 6 weeks of birth. Ignorance regarding clubfoot and its treatment may be a factor leading to late presentations. Although there are no previous data for comparison, the number of children requiring casts and the age at which treatment is initiated have definitely changed over the last three years. During the first year there were 5 patients (22.7%) who were less than 6 months of age, this number improved to 12 patients (30.7%) in the second year which further improved to 41 (53.2%) in the third year. The improvement in the number and percentages clearly reflect the change. This change may be due to the various awareness campaigns that were undertaken by the NGO, their link-ups with other NGOs dealing with similar issues, and their promotion of the clubfoot clinic through print media in various private and government hospitals of the region.

Non-governmental organizations can play an important role in this direction. They can act as a bridge between the hospital and the community by mobilizing people, taking collective action, organizing public awareness programs, and representing the interests of the patients in the hospital. The response to Question 9 (patient-counselor bonding) reflected the bonding that the parents make with the counselors. Parents had given a mean score of 8.89 and the observers had given a mean score of 8.79. The parents were able to connect with the counselors and identify them as a part of the society to which they belong. The counselors often use local dialect and have knowledge of common issues and frequent problems faced by the parents both in the society and the hospital. Their feedback helps in streamlining our functioning for running the clinic smoothly.

The average response given by parents for the role of NGO was significantly higher than observers’ response (*p* < 0.001), indicating that the parents perceived a greater benefit from the functioning of the NGO. Parents received a helping hand from the NGO throughout the treatment process, from the initial registration at the clinic to the application of braces. The NGO supported the parents in scheduling their visit in the clinic, reminding them of their follow-up visits, counseling them about clubfoot, and providing feedback to the doctors to streamline the functioning of the clinic. The common concerns about treatment in tertiary centers like delay, negligence, inadequate communication, and feelings of isolation or being marginalized were neutralized to a great extent, and this may have contributed to a favorable response from the parents for the NGO when compared with the observers’ response.

The number of casts per foot in our study was four to 10 (average 6.6). In a study by Ponseti and Smoley, the number of cast per feet was five to 10 (average 7.6) [6]. In another study by Laaveg and Ponseti, the mean number of casts during their treatment was seven [7]. Morcuende et al. reported that 90% of the patients required five or fewer casts [8, 9]. In our study, five patients (3.7%) required extensive surgical correction; this was similar to the data (2.5%) reported by Morcuende et al. [9]. Relapse rate in the patients treated by casting was 7.5% in our study, which was also similar to other reported studies [8, 9]. There was a 93.3% success rate of correction of clubfoot at our clinic, which was better than the 88.5% success rate reported by Laaveg and Ponseti [7], but less than 98.0% reported by Morcuende et al. [9]. Regular documentation of the Pirani scores and weekly follow-up of patients helped in keeping up with the standards of treatment and providing results comparable to other studies.

Some of the limitations of our study include the relatively small sample size and the short period of data collection, which may have precluded our ability to observe rare but important safety concerns or potential institutional resistance. In addition, the awareness that they were being assessed might have impacted the performance of the NGO. Because this study was a site-specific, observational study, in a clinic that benefits from additional NGO resources, results may not be applicable to other settings.

There were a few concerns with respect to the working of the NGO at our clinic. Owing to limited training, NGOs may not be in a position to conduct top quality research, and scientific rigor may be lacking in certain instances. Lack of understanding of scientific literature can also be a major shortfall. The relationship between NGOs and government may involve political, legal, ideological, and administrative constraints. Because of their voluntary nature, there may be questions regarding the legitimacy, accountability, and credibility of NGOs. Questions may also be asked about the motivations and objectives of NGOs, and the degree of accountability they accept for the ultimate impact of the policies and positions they advocate.

## Conclusion

Despite all concerns and limitations, our study illustrates that the NGO associated with our clubfoot clinic successfully supported formal health care professionals and helped reduce their daily workload. Its association with the clinic has helped in streamlining its functioning. From receiving the patients to their final cast application, the NGO maintained regular contact with them and their parents, and has emerged as a very effective tool in successful running of the clinic.

Further investigation into the expansion and scope for new roles and tasks performed by such NGOs should be undertaken to analyze their role on a larger scale in clubfoot program.

## Conflict of interest

PS, RV, AG and SG declare no conflict of interest in relation with this paper.

## Appendix I

### Questionnaire

Name of the Patient/Observer :

Date/Time :

Give the response to each question on a scale of 1 to 10

1 : Worst

10 : Best

**Table:**

How do you grade the:		
1.	Availability of the counselor	___________________
2.	Duration of counseling sessions	___________________
3.	Content of the counseling	___________________
4.	Quality of study material provided	___________________
5.	Record keeping of child	___________________
6.	Role in ensuring follow-up	___________________
7.	Help provided during visit to the hospital	___________________
8.	Quality of braces provided	___________________
9.	Patient counselor bonding	___________________
10.	Reputation of the NGO among parents of children with clubfoot	___________________
